# Osteosarcoma progression is associated with increased nuclear levels and transcriptional activity of activated β-Catenin

**DOI:** 10.18632/genesandcancer.191

**Published:** 2019-05

**Authors:** Noureen Ali, Geetha Venkateswaran, Elizabeth Garcia, Takaaki Landry, Hunter McColl, Consolato Sergi, Amit Persad, Yasser Abuetabh, David D. Eisenstat, Sujata Persad

**Affiliations:** ^1^ Department of Pediatrics, University of Alberta, Edmonton, Alberta, Canada; ^2^ Department of Medical Genetics, University of Alberta, Edmonton, Alberta, Canada; ^3^ Department of Oncology, University of Alberta, Edmonton, Alberta, Canada; ^4^ Department of Laboratory Medicine & Pathology, University of Alberta, Edmonton, Alberta, Canada

**Keywords:** ABC, β-catenin, osteosarcoma progression, transcription, biomarker

## Abstract

Osteosarcoma (OS) is an aggressive primary bone malignancy that has peak incidence in children and young adults <25 years of age. Despite current multimodal treatments, no significant change in patient outcome has been observed in two decades. Presently, there is a lack of established, reliable baseline prognostic markers for aggressive OS, other than extent and site of disease involvement.

The canonical Wnt/β-catenin pathway controls multiple cellular processes, and is known to be a critical pathway in OS progression. This pathway regulates cellular levels of β-catenin, which is a significant player in the oncogenesis and progression of many cancers.

We investigated the relationship between β-catenin, more specifically, the transcriptionally active form of β-catenin, Activated β-Catenin (ABC), and OS progression. Using an *in vitro* model, we observed that cellular/nuclear ABC levels, but not cellular/nuclear β-catenin levels, increase with the degree of aggressiveness in OS. Our results demonstrate a strong association between nuclear-ABC levels and aggressive OS *in vitro*. Furthermore, we observed significant correlation between positive nuclear-ABC and patient age and tumor stage.

Our results support the potential use of ABC as a predictive marker for risk stratification in OS.

## INTRODUCTION

Osteosarcomas (OS) are aggressive primary bone malignancies that have peak incidence in children and young adults < 25 years of age [1–6]. The 5-year event-free survival (EFS) rate for patients with localized disease is approximately 70% [1–7]. In contrast, outcome remains poor for most patients with metastatic disease, with a 5 years EFS rate of approximately 20% [1, 8–13]. Metastatic disease progression occurs in 1 of 5 cases of localized OS despite intensive chemotherapy [1, 7, 10, 14–19]. Approximately, 20% of patients present with metastatic dissemination (usually lungs) at diagnosis [1, 13]. Presently, there are no widely recommended screening tests for early diagnosis of OS and no reliable baseline prognostic marker for aggressive OS that have been established. This highlights the need for identifying prognostic markers that would facilitate risk stratification and identify the patient groups in which novel therapeutic interventions are most needed. Therefore, it is crucial to understand the pathobiology of OS progression in order to identify clinically reliable prognostic markers for OS.

The Wnt/β-catenin signaling has been shown to be an “oncogenic” pathway that promotes tumor progression in many cancers. β-catenin is the central transcriptional mediator of the canonical *Wnt* signaling pathway, which is aberrantly regulated in many adult and pediatric cancers [20]. β-catenin plays a role in intracellular adhesion and in regulating gene expression. The latter role is associated with its oncogenic properties. β-catenin is a transcriptional/transactivating factor serving as a co-activator of the lymphoid enhancer factor/T-cell factor (TCF) family of DNA-binding proteins [21, 22]. Binding of the TCF/β-catenin bipartite complex on TCF binding sites activates transcription of genes that control cell proliferation, differentiation and invasiveness.

Although cytoplasmic accumulation of β-catenin and its subsequent translocation to the nucleus is thought to induce β-catenin/TCF transcriptional activity, the transcriptionally active form of β-catenin, Active β-Catenin (ABC), which is dephosphorylated at Serine 37 and Threonine 41 at the N-terminal domain of the protein, constitutes a unique pool of β-catenin [23, 24]. This form of activated β-catenin or ABC, is recognized by a monoclonal antibody (mAb 8E7, Millipore Sigma) only when Serine 37 and Threonine 41 at the N-terminal domain of the protein are not phosphorylated. ABC is largely monomeric, located mainly within the nucleus and found to be intrinsically more transcriptionally active than the non-phosphorylated β-catenin [24]. High levels of nuclear ABC compared to unphosphorylated total cellular β-catenin are associated with significantly worse overall survival [25]. However, it should be clarified that ABC can also interact with E-cadherin at the cell membrane where it is not transcriptionally active [26].

The canonical Wnt/β-catenin pathway plays an important role in regulating bone remodeling [27] and is dysregulated in OS. Increase in the expression of LRP5, a co-receptor in the Wnt pathway, correlates with OS metastasis [28], while blocking LRP5 was shown to decrease invasiveness of OS [29, 30]. Furthermore, epigenetic silencing of the Wnt antagonist, Wnt inhibitory factor (WIF) is observed in OS [31] and ectopic expression of Wnt antagonists, WIF and Dickopff (Dkk) have been shown to attenuate the tumorigenicity and metastatic potential of OS [32–34]. While these studies are supportive of the role of canonical Wnt signaling in OS pathobiology, studies that focus on the role of β-catenin in OS provides mixed evidence. Based on immunohistochemical (IHC) analysis of β-catenin on patient samples and OS cell lines, some studies report a correlation between cytoplasmic/nuclear staining of β-catenin and lung metastasis [22–24, 35], whereas others report lack of nuclear staining of β-catenin in OS patient samples and cell lines [36, 37]. However, these studies focused on localization of β-catenin rather than the transcriptionally active ABC. Moreover, these studies did not address the functional role of β-catenin in OS progression.

In this study, we used human OS cell lines to investigate the putative relationship between β-catenin, more specifically ABC, and OS progression with the future goal of understanding whether ABC can be used as a biomarker of risk stratification in OS patients. Our results show that while the levels of cellular and nuclear β-catenin remain unaltered, cellular and nuclear levels of ABC increase with OS progression. Our results suggest that Wnt pathway's role in mediating OS progression is regulated specifically through ABC and not β-catenin.

## RESULTS

### Confirmation of the metastatic potential in OS cell lines

**Figure 1:**
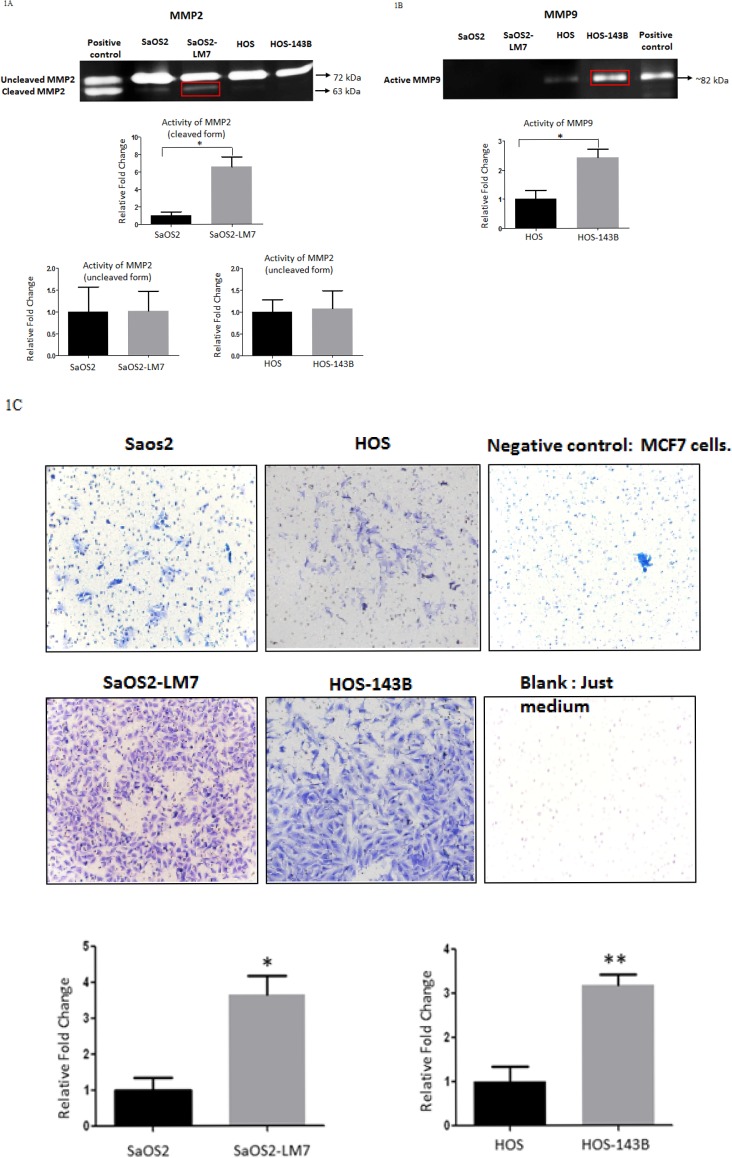
Comparison of potential aggressive/metastatic phenotype of OS cell lines *in vitro* Gelatin zymography was used as a measure of the activity for **A.** MMP2 and **B.** MMP9 in concentrated conditioned media from cells grown in serum free media for 24 h. No significant difference was observed in the activity of the un-cleaved form of MMP2 in either pair of cell lines. However, there was a significantly greater activity observed in the cleaved MMP2 in the SaOS2-LM7 cell line compared to SaOS2. Data is representative data of 6 or more experiments; ^*^p<0.05. Although HOS/HOS-143B did not show any activity for cleaved MMP2, there was significantly greater MMP9 activity in the HOS-143B cells compared to HOS cells. Data is representative data of 6 or more experiments; ^***^p < 0.001. The SaOS2/SaOS2-LM7 pair did not exhibit any MMP9 activity. **C.** Figure shows comparison of the invasive potential of the two paired OS cell lines (SaOS2/SaOS2-LM7 and HOS/HOS-143B using Transwell invasion assay through Matrigel. We observed significantly higher numbers of invaded cells for SaOS2-LM7 and HOS-143B compared to SaOS2 and HOS cells, respectively. Each group of data is representative of 3 or more experiments. ^*^p < 0.05; ^**^p < 0.01. The upper panels show representative images of the invaded cells.

We used two pairs of human OS cell lines, each pair consisting of a parent cell line and a metastatic cell line derived from the parent cell line: pair 1) SaOS2 (parent cell line) and SaOS2-LM7 (metastatic cell line); pair 2) HOS (parent cell line) and HOS-143B (metastatic cell line). SaOS2-LM7 and HOS-143B cell lines exhibit greater metastatic potential compared to their respective parent cell lines SaOS2 and HOS [38, 39]. We compared and confirmed the metastatic potential of these cell lines by MMP2 and MMP9 activity using gelatin zymography. Several studies on clinical samples of OS patients have suggested an increased expression of MMP2 and MMP9 in high grade OS [40, 41]. Our results showed that the gelatinase activity of the pro-enzyme form or inactive form of MMP2 (72 kDa) was similar in each pair of the two paired cell lines (Figure 1A). However, the gelatinase activity of the active form of MMP2 (63 kDa) was significantly greater in SaOS2-LM7 compared to its parent cell line SaOS2 (Figure 1A). In contrast, the gelatinase activity of MMP9 (82 kDa) was significantly greater in HOS-143B compared to the parent cell line HOS (Figure 1B). There was no measurable MMP9 activity in SaOS2/ SaOS2-LM7 cells and we did not observe any measurable MMP2 activity in the HOS/HOS 143B cell pair.

We further confirmed the metastatic potential of the two paired OS cell lines using the Matrigel invasion assay. Non-invasive breast cancer cell line MCF-7 was used as a negative control and alone without any cells was used as a blank. As shown in Figure 1C, that invasiveness was significantly higher in of the SaOS2-LM7 and HOS-143B cell lines compared to the parental SaOS2 and HOS cells, respectively. This corroborates with the higher MMP2 activity in SaOS2-LM7 compared to SaOS2 (Figure 1A) and the significantly higher activity of active MMP9 seen in HOS-143B compared to HOS cells (Figure 1B). These results support the use of the SaOS2/SaOS2-LM7 and HOS/HOS-143B paired of OS cell lines as a useful effective model for OS progression.

### Cellular and nuclear levels of ABC increase with OS progression

**Figure 2:**
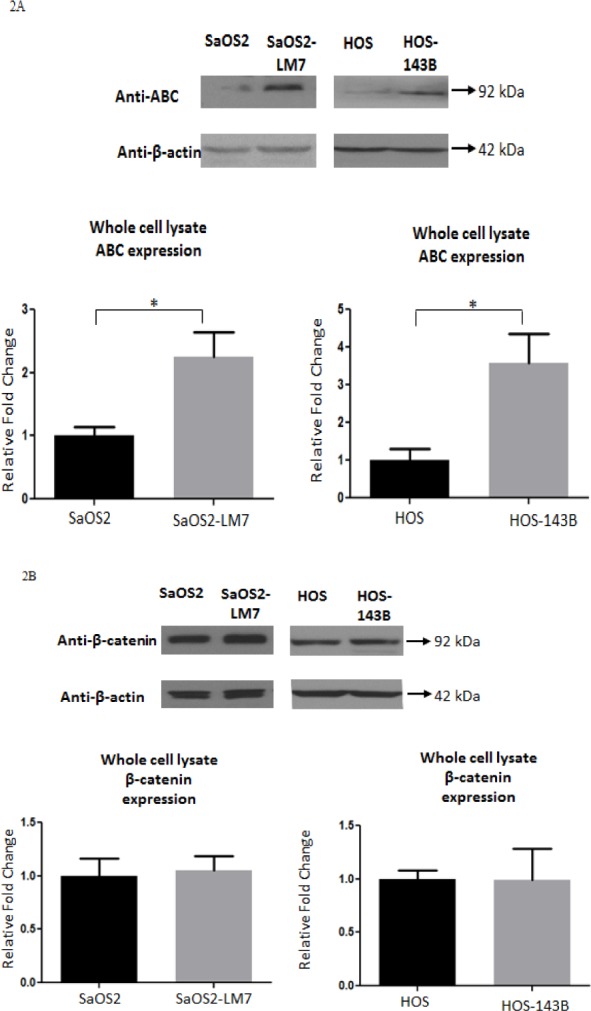
Cellular level of ABC increases with OS progression Western blot analysis of the levels of **A.** ABC and **B.** β-Catenin in whole cell lysate of OS cell lines. The cellular level of ABC was observed to be significantly higher in the SaOS2-LM7 and HOS-143B cell lines compared to their parent cell lines, SaOS2 and HOS, respectively. There were no differences in β-catenin levels between the SaOS2/ SaOS2-LM7 pair and HOS/HOS-143B pair of OS cell lines. Data is representative of 6 or more experiments; ^*^p < 0.05.

To determine whether cellular levels of β-catenin and ABC are altered with OS progression, we carried out Western blot analysis for β-catenin and ABC on total cellular lysates from the two paired cell lines. Our results showed that the total cellular levels of ABC were significantly greater in the two metastatic cell lines (SaOS2-LM7 and HOS-143B) compared to their parent cell lines (SaOS2 and HOS) (Figure 2A). On the other hand, the total cellular levels of β-catenin were unaltered in the metastatic cell lines compared to their parent cell lines (Figure 2B).

**Figure 3:**
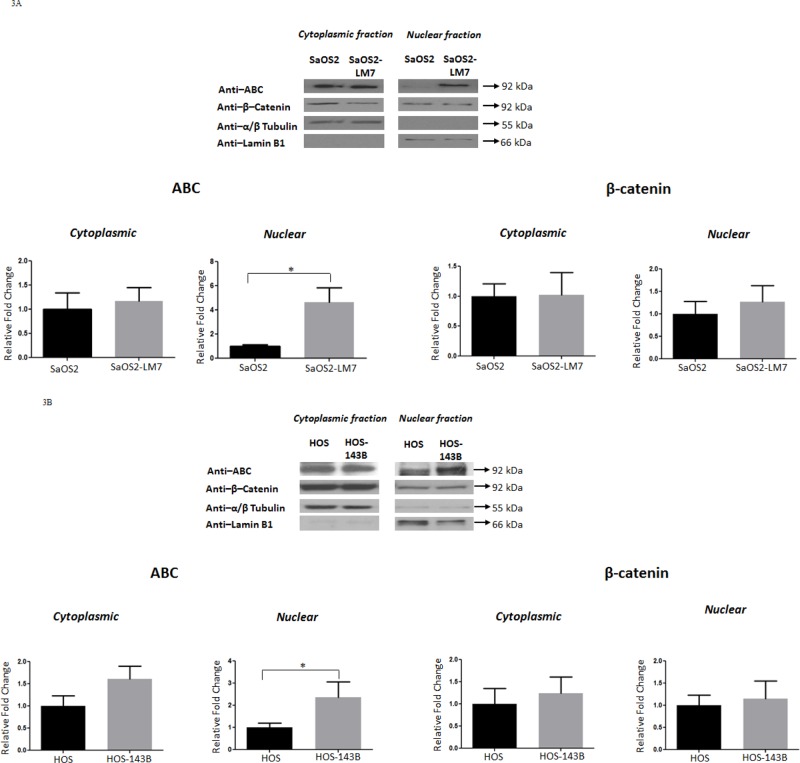
Nuclear levels of ABC increases with OS progression Western blot analysis for ABC and β-catenin in cytoplasmic and nuclear fractions of **A.** SaOS2 and SaOS2-LM7 cell lines and **B.** HOS and HOS-143B cell lines. Nuclear ABC levels were significantly higher in SaOS2-LM7 and HOS-143B cells compared to SaOS2 and HOS cells, respectively. No differences were observed in the levels of cytoplasmic ABC or cytoplasmic and nuclear β-catenin. Data is representative of 6 or more experiments. ^*^p < 0.05.

Since β-catenin is a protein that shuttles between the cytoplasm and nucleus, and its oncogenic/transcriptional role is executed within the nucleus, we next determined the subcellular distribution/levels of β-catenin and ABC (cytoplasmic and nuclear). Western blot analysis for β-catenin and ABC on the cytoplasmic and nuclear fractions isolated from the two paired cell lines showed a higher level of ABC in the nuclear fraction of SaOS2-LM7 and HOS-143B compared to SaOS2 and HOS, respectively. However, there was no difference in the levels of total β-catenin in the nuclear fractions. Further, there was no significant difference in the level of β-catenin or ABC in the cytoplasmic fractions of either pair of cell lines (Figure 3A and 3B). Therefore, our data indicates that while nuclear ABC level alterations are strongly associated with OS progression, nuclear β-catenin is not. α/β-tubulin and Lamin B1 were used as cytoplasmic and nuclear markers, respectively, to indicate the relative enrichment of these two subcellular fractions prior to immunoblotting.

**Figure 4:**
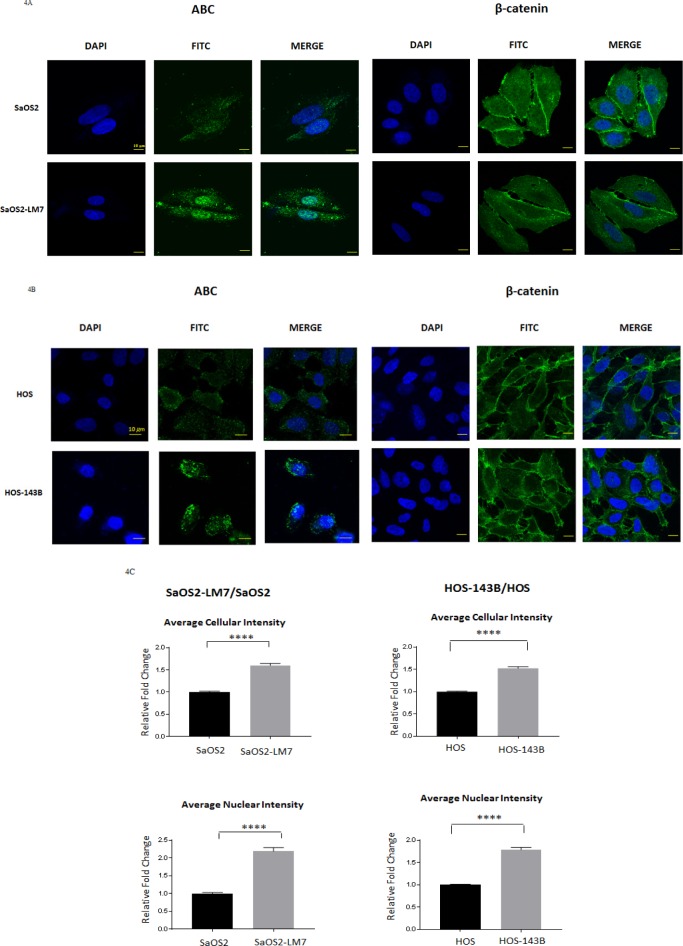
Cellular localization and quantification of ABC fluorescence intensity in OS cell lines Immunofluorescence analysis for ABC and β-Catenin in **A.** SaOS2 and SaOS2-LM7 cells and **b.** HOS and HOS-143B cells. Figure shows higher levels of ABC in SaOS2-LM7 and HOS-143B cells that is predominantly nuclear compared to SaOS2 and HOS, respectively. There was no difference observed in the cellular localization of β-Catenin in the SaOS2/SaOS2-LM7 or HOS/HOS-143B cell pairs. IF images are representative of 6 or more experiments. **C.** Quantification of total cellular and nuclear levels of ABC in SaOS2/SaOS2-LM7 and HOS/HOS-143B cells (from IF images (10X) using MetaXpress software). Results show that there were significantly higher levels of ABC in the cytoplasm, and more importantly in the nucleus, of the SaOS2-LM7 and HOS-143B cells compared to their parental lines. Each group of data is representative of 6 or more experiments. ^***^p < 0.0001.

Differences in cellular levels and subcellular localization of β-catenin and ABC were further investigated using immunofluorescence (IF) analysis in the two paired OS cell lines. IF images showed a predominant nuclear localization of ABC which was significantly more intense in both SaOS2-LM7 (Figure 4A) and HOS-143B (Figure 4B) cells when compared to their respective parental cell lines. On the other hand, β-catenin showed strong cytoplasmic and membranous localization, with little difference in β-catenin levels between each paired cell lines (Figure 4A and 4B).

To further quantify our observations with respect to total cellular and the nuclear intensity of ABC signal in both pairs of OS cell lines, we carried out high content analysis of ABC immunofluorescence using MetaXpress software. The high content analysis data showed that both total cellular and nuclear intensity of ABC were significantly higher in the metastatic cell lines (SaOS2-LM7 and HOS-143B) compared to the parent cell lines (Figure 4C). Collectively, these results are in concordance with the Western blot analysis and suggest that while increased cellular/nuclear expression of ABC is significantly associated with OS progression, cellular/ nuclear levels of β-catenin are not associated with OS progression.

### The mRNA expression of ABC target genes increases with OS progression

**Figure 5:**
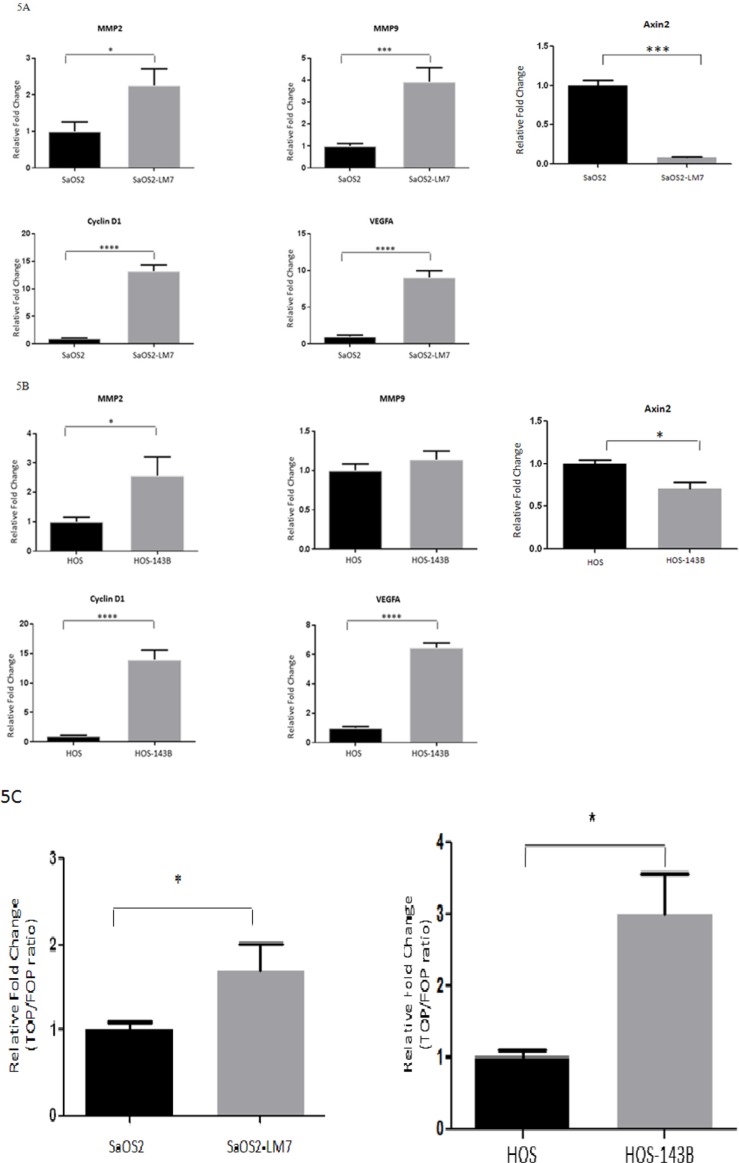
Transcriptional activity of ABC/β-catenin increases with OS progression mRNA expression of Wnt/β-catenin target genes (MMP2, MMP9, Cyclin D1, VEGF-A and Axin2 in: **A.** SaOS2 and SaOS2-LM7 cell lines and **B.** HOS and HOS-143B cell lines. Target gene expression was quantified by RT-qPCR and shows that mRNA expression of MMP2, MMP9, Cyclin D1, VEGF-A was higher in the SaOS2-LM7 and HOS-143B cell lines compared to the parental SaOS2 or HOS cells, respectively. On the other hand, mRNA expression of Axin2 was significantly lower in the SaOS2-LM7 and HOS-143B cell lines compared to the parental SaOS2 or HOS cells, respectively. Each group of data is representative of 4 or more experiments. ^*^p < 0.05; ^***^p < 0.0005. **C.** -catenin/TCF transcriptional activity as measured by TopFlash reporter assay were higher in the HOS-143B and SaOS2-LM7 cells compared to parental HOS and SaOS2 cells, respectively. Each group of data is representative of 6 or more experiments. ^*^p < 0.001; ^**^p < 0.0001.

β-catenin regulates the transcriptional expression of numerous target genes through its interaction with TCF-4, including Cyclin D1, VEGFA, MMP2, MMP9 and Axin2 [42–44]. We examined whether the mRNA expression of Cyclin D1, VEGFA, MMP2, MMP9 and Axin2 was altered with OS progression. Quantitative RT-qPCR analysis of total RNA from the SaOS2 LM7/SaOS2 paired cell lines indicated that there was a significant increase in mRNA expression of VEGFA (9.06 fold), Cyclin D1 (13.2 fold), MMP2 (2.5 fold), MMP9 (4.0 fold) and a significant decrease in Axin2 (0.1 fold) in SaOS2-LM7 compared to SaOS2 cells (Figure 5A). Similarly, for the HOS-143B/ HOS paired cell lines, the mRNA expressions of VEGFA (6.5 fold), Cyclin D1 (13.96 fold) and MMP2 (2.7 fold) in the HOS-143B cells was significantly higher, while Axin2 levels were significantly lower (0.6 fold) compared to the HOS cells (Figure 5B). However, we did not observe any difference in the mRNA expression of MMP9 between HOS and HOS-143B cells (Figure 5B), which differs from our findings that gelatinase activity of MMP9 is significantly higher in HOS-143B compared to the parental HOS cells. Furthermore, while both SaOS2 and SaOS2-LM7 exhibited significant mRNA expression of MMP9 with higher expression in SaOS2-LM7 compared to SaOS2, we did not observe any measurable MMP9 activity in either cell line. Despite these discrepancies between MMP9 gene expression and enzymatic activity, overall these quantitative gene expression results provide further support that the TCF/LEF transcriptional activity increases with OS progression.

### TCF/LEF transcriptional activity increases with OS progression

We further investigated whether the higher ABC levels in the more metastatic cell lines is functionally demonstrated by an increased transcriptional activity *in vitro*. We monitored transcriptional activity by measuring TOPFlash-luciferase reporter activity in the HOS/HOS-143B and SaOS2/SaOS2-LM7 paired cell lines. Our findings show that TOPFlash luciferase activity was significantly higher in the HOS-143B (2.99 fold) and SaOS2-LM7 (1.7 fold) cell lines compared to the HOS and SaOS cells, respectively (Figure 5C). This fully correlates with the higher nuclear levels of ABC (Figure 3) and higher mRNA levels of key target genes (Figure 5B) that we observed in the metastatic lines compared to their parental cell lines.

### Immunohistochemical staining of OS patient tissue and OS TMA with ABC

**Figure 6:**
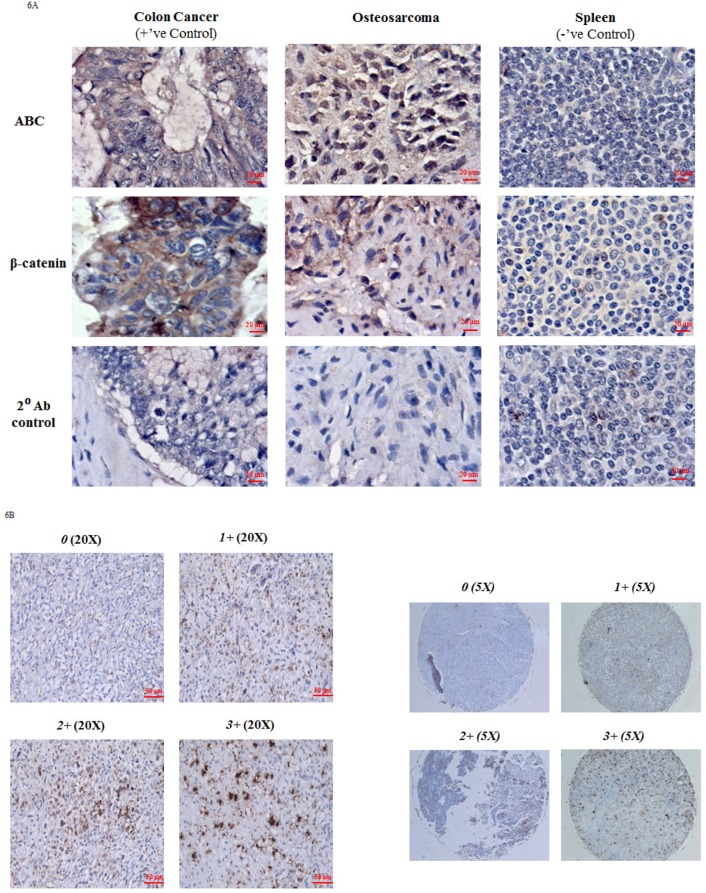
Immunohistochemical analysis of ABC and β-catenin in OS tissue **A.** Figure shows immunoexpression of ABC and β-catenin in OS tissue. Colon cancer tissue served as a positive tissue control and spleen tissue was used as negative tissue control. Immunoexpression data is representative of 6 experiments. **B.** Representative data of nuclear ABC protein staining in osteosarcoma tissue cores on TMA slide at magnification 20X and 5X (entire core). 0 (no nuclear staining); 1+ (≤30%); 2+ (31-60%); 3+ (>60%).

Results from the *in vitro* cell culture-based analysis suggest that ABC levels and transcriptional activity are directly associated with OS progression. To explore the clinical relevance of our findings, we carried out immunohistochemical analysis (IHC) of ABC and β-catenin in OS tissue samples. Colon cancer tissue was used as a positive control and exhibited strong IHC staining for both β-catenin and ABC at the colonic crypts. Spleen was used as a negative tissue control and showed no staining for β-catenin or ABC. All OS tissue stained for IHC showed extensive staining for both β-catenin and ABC (Figure 6A). However, while the ABC staining was mainly localized to the nuclei, the β-catenin staining was observed to be mainly localized to the extra-nuclear regions (Figure 6A).

We further confirmed the nuclear localization of ABC by using a commercially available OS tumor tissue microarray (TMA) which was comprised of 40 embedded histologically confirmed primary OS samples (adults and children) each in duplicate (Folio BioSci). The patient information provided by the company was limited and included information on stage of the tumors, age and sex of patients but not patient outcomes. Figure 6B shows OS tumor tissue cores with ABC nuclear staining. ABC nuclear staining, of various staining intensities, was detected in 34 (85%) out of 40 tumor cores (Table 1). 6 (15%) out of 40 tumor cores were negative for nuclear ABC staining. Among the positive tumors, 29 (85%) showed 1+ (≤ 30% positive nuclear staining cells), 3 tumors (10.30%) showed 2+ (31% -60% positive nuclear staining cells) and 2 tumors (7%) had 3+ (61%-100% positive nuclear staining) staining. The representative cores were from tumors of stages IA, IB, IIA and IIB.

**Table 1:** Summary of clinicopathologic data and ABC immunostaining in osteosarcoma tumor tissue microarray

	Total	ABC positive nuclear staining			ABC negative nuclear staining	*P* value
Total	40	34 (85%)			6 (15%)	
Gender						
Male	27	22 (81.5%)			5 (18.5%)	0.64^a^
Female	13	12 (92.3%)			1 (7.7%)	
Mean Age (years)		28.91			44.67	0.01^b^
Scores/Tumor Stage		1+	2+	3+	0	
IA	2	1	0	0	1	0.04^c^
IB	3	1	1	0	1	
IIA	9	6	0	1	2	
IIB	26	21	2	1	2	

Table summarizes that there is significant correlation between positive ABC nuclear staining intensity and age of patient and tumor stage. There is no correlation between positive ABC nuclear staining intensity and gender. Statistical analysis for gender, age, and tumor stage was done using unpaired student's *t*-test, non-parametric Mann-Whitney test and Kruskal-Wallis trend test respectively; *P* < 0.05.

a Indicates the *P* value for nuclear ABC staining in males *vs* females.

b Indicates the *P* value for difference between mean ages of patients in positive and negative ABC staining groups.

c Indicates the *P* value for nuclear ABC staining across different stages of OS tumor (IA, IB, IIA & IIB).

There was no significant correlation between gender of patients and nuclear ABC staining as evaluated by Fisher's exact test (*p* = 0.64). However, we found that patients with positive nuclear ABC staining were, on average, younger (mean age = 28.91 years) than patients with negative nuclear ABC staining (mean age = 44.67 years). Statistical analysis, using non-parametric Mann-Whitney test, showed that the correlation between average age and positive ABC nuclear staining was significant (*p* = 0.01). We also examined the relationship between positive ABC nuclear staining and OS tumor stage. Positive ABC nuclear staining was present in 24 of 26 (92%) stage IIB OS tumors, 7 of 9 (77%) of the stage IIA tumors, 2 of 3 (66%) of the stage IB tumors and 1 of 2 (50%) of the stage IA tumors. Correlation between ABC nuclear staining and tumor stage was statistically tested using the Kruskal Wallis Trend test and showed that positive nuclear ABC staining was significantly (*p* = 0.04) correlated with stage of tumor.

## DISCUSSION

The Wnt/β-catenin pathway plays a crucial role in skeletal development and is indispensable for osteoblast lineage determination [27]. Presently known molecular events underlying the genesis and progression of OS have highlighted that Wnt signaling is a significant pathway in the pathogenesis of OS. Although previous studies have shown deregulation of Wnt signaling pathway in OS [28–34], the role of β-catenin itself in OS and more specifically in OS progression remains unclear. In the few studies that have investigated the role of β-catenin in OS, the main focus has been on the cellular localization of total β-catenin with findings that are inconclusive and controversial. Staal et al (2001) showed that the cellular/ nuclear accumulation of β-catenin is not sufficient to transduce Wnt signals. However, increase in nuclear levels of the N-terminally dephosphorylated form of β-catenin, Active β-Catenin (ABC), is a significant component that constitutes the key oncogenic step [23]. In our study, we used two pairs of human OS cell lines, SaOS2/SaOS2-LM7 and HOS/HOS-143B and human OS tissue, to investigate the putative relationship between ABC/β-catenin and OS progression. To the best of our knowledge, the role of ABC in OS pathogenesis/ progression has never been reported. Our results suggest that cellular levels and subcellular localization of ABC were significantly associated with OS progression and an aggressive OS phenotype *in vitro*. However, cellular levels and subcellular localization of total β-catenin were not significantly associated with OS progression.

We confirmed the metastatic potential of the OS cell lines using matrigel invasion assay and gelatin zymography to measure MMP2 and MMP9 activity. MMP2 and MMP9 activity has been previously shown to be directly associated with high grade OS [40, 41]. Our results showed higher activity of active MMP2 in SaOS2-LM7 compared to SaOS2, whereas MMP9 activity was higher in the metastatic cell line HOS-143B compared to the HOS cell line (Figure 1). These results corroborated completely with the higher invasiveness of the SaOS2-LM7 and HOS-143B cell lines compared to the parental SaOS2 and HOS cells, respectively (Figure 1C) and supported our use of these paired OS cell lines (SaOS2/ SaOS2-LM7 and HOS/HOS-143B) as appropriate models for studying OS progression.

Using these paired cell lines, we show that cellular, and more specifically, nuclear levels of ABC were higher in the more aggressive OS cell lines (SaOS2-LM7 and HOS-143B) compared to the less aggressive parental lines (SaOS2 and HOS). However, there was no difference observed in cellular/nuclear β-catenin levels within each pair of cell lines (Figure 2 & 3). The results from IF analysis of cellular levels and localization of ABC and β-catenin corresponded with the Western blot data (Figure 4A & 4B). This was further supported by quantification of the IF data with high content microscopy (10X) which showed greater total cellular and nuclear intensity of the IF signal for ABC in SaOS2-LM7/HOS-143B cells compared to parental SaOS2/HOS, respectively (Figure 4C). Our results indicate that cellular, and more importantly nuclear, ABC levels are higher in metastatic OS cell lines compared to the respective parental cell lines. Correlating with higher nuclear ABC levels, β-catenin/TCF-specific transcriptional activity, measured by TopFlash reporter activity assay, was significantly higher in SaOS2-LM7 and HOS-143B cells compared to the parental SaOS2 and HOS cells, respectively (Figure 5C). Since we did not observe any significant difference in cellular/nuclear β-catenin levels in the paired cell lines, the higher TopFlash activity in SaOS2-LM7 and HOS-143B cells likely corresponds to the higher nuclear ABC levels in these cells compared to the parental cells. The greater transcriptional activity in the more aggressive OS cells is further supported by the higher mRNA expressions of target genes of ABC transcriptional activity, namely MMP2, MMP9, Cyclin D1 and VEGFA (Figure 5A & 5B) in the more metastatic cell lines compared to the parental cell lines. It should be pointed out that the gene expression changes of MMP2 and MMP9 shown in Figures 5A and 5B does not correlate stringently with the enzyme activities shown in Figure 1A and 1B. This can be explained by the fact that mRNA expressions are not always directly proportional to protein levels or activity. Hence, an increased/decreased mRNA expression may not always be represented by an equivalent/parallel change in protein levels or activity.

Interestingly, we observed a significant decrease in Axin2 mRNA levels in the SaOS2-LM7 and HOS-143B cell lines compared to the respective parental cell lines (Figure 5A & 5B). Axin2 is a tumor suppressor that is induced upon activation of Wnt/beta-catenin pathway. Axin2 then acts as a negative feedback regulator of the canonical Wnt pathway activation and effectively down-regulates cellular/nuclear β-catenin levels thus keeping the pathway under tight control [43, 44]. However, it is known that Wnt/beta-catenin target genes can also be regulated by other cellular signals which may take a more dominant role in regulating these genes under certain contexts [26, 44]. Such a phenomenon may be functional in inducing a decrease in Axin2 expression that we observe. In support of this, Lu et al. showed that the micro RNA, miR-374a, which is increased in osteosarcoma tissue, directly targeted Axin2, suppressed its activity and promoted growth of osteosarcoma cells. The existence of negative feedback loops (Axin2) are well accepted to be highly essential, under normal circumstances, to correctly regulate/control the activity of pathways such as the canonical Wnt pathway. However, other mechanisms/ pathways may override these controls under disease conditions, such as cancer, in order to promote disease progression (tumorigenicity, tumor growth and tumor progression). This is a likely reason why we observed a decrease in Axin2 expression in the more aggressive cell lines (SaOS2-LM7 and HOS-143B) compared to their less aggressive parental lines.

We also noted that the increase in TCF-mediated transcriptional activity, as shown by TopFlash reporter assay and mRNA expression of target genes are significantly more robust (4-15 fold) in comparison to the fold change observed with cellular/nuclear ABC levels. This phenomenon, where modest alterations in ABC result in significant transcriptional efficacy, has been previously reported [23]. It was suggested that the phosphorylation status of ABC may play a key role in the efficacy with which ABC is imported to the nucleus [23]. It is also possible that the phosphorylation status of ABC positively emphasizes its efficiency as a transcriptional regulator by augmenting its interaction with TCF transcription factor and/or other co-factors. However, this is not presently known. Nonetheless, our present data supports that nuclear ABC plays a significant role in promoting the metastatic potential of OS as determined by the analysis of OS cell lines *in vitro*.

Our results highlighting the significance of cellular/ nuclear ABC levels, and not β-catenin, in OS progression/ aggressiveness, may explain the conflicting reports in the present literature over the impact of the Wnt/β-catenin pathway on OS pathogenesis. Although there is significant evidence for the involvement of the Wnt signaling pathway in OS, there are only a few studies that have investigated the specific role of β-catenin in OS progression and their findings are disparate. These studies have investigated the cellular localization of β-catenin *in vitro, in vivo* and on clinical samples of OS. Iwaya *et al*, using a murine model of OS, proposed that there was increased level of nuclear β-catenin associated with OS progression [35], while Kidani *et al,* in contrast, showed decreased metastatic potential with increased cytoplasmic β-catenin [45]. Overall, the role of β-catenin in OS progression using murine models has been largely inconclusive. Further, a study by Cai *et al*, using a panel of human OS cell lines, suggested that there was no positive association between nuclear β-catenin accumulation and OS progression [37]. Similarly, studies carried out using OS patient samples have also reported contradictory findings with regards to the association of β-catenin in OS progression. A study by Haydon *et al*, showed cytoplasmic and/or nuclear accumulation of β-catenin in 70% of OS patients [22]. However, it was later revealed that only 3 among 47 patients in the study presented with positive nuclear β-catenin staining [36]. Correlation between β-catenin staining and the corresponding available clinic-pathologic data showed accumulation of β-catenin to be a common event in OS, but not significantly associated with OS progression [22, 36]. In support of this observation, another study by Du *et al*, showed no nuclear β-catenin staining in 46 osteosarcoma samples tested, but 32 of 46 showed cytoplasmic and membranous β-catenin staining [46]. In contrast, a study by Lu *et al*, reported cytoplasmic β-catenin staining in 66 of 96 OS cases, with correlational analysis indicating that aberrant β-catenin expression was significantly associated with metastasis and decreased patient survival. However, it is important to note that this study did not measure nuclear β-catenin levels, the subcellular organelle most relevant to β-catenin's oncogenic properties [47]. These studies suggest that while β-catenin expression is evident in OS, it may not be associated with or of relevance to OS progression. In concordance with these reports, our study also found that cellular levels and localization of β-catenin was unaltered with OS progression and likely not significant in OS pathogenesis/aggressiveness. Our data is also in agreement with the findings of Staal et al [23] which states that it is not β-catenin but its dephosphorylated active form, ABC, the focus of our study, that is significant in promoting oncogenic potential.

In order to characterize the relationship between the protein expresssion of ABC and clinical and demographic variables, we used a commercially available OS tumor tissue microarray (TMA) which comprised of 40 embedded histologically confirmed primary OS samples in duplicate and included information on age, gender and stage of the tumors. ABC nuclear staining was detected in 34 (85%) out of 40 tumor cores (Table 1). The representative cores were from tumors of stages IA, IB, IIA and IIB. Statistical analysis showed that there was significant (*p* = 0.04) association between tumor stage and positive nuclear ABC staining. Interestingly, statistical analysis also showed that, on average, positive nuclear ABC staining was seen mainly in tumors from younger patients (*p* = 0.01). In this respect it is of importance to note that all ABC negative tumors in our cohort were from adult patients and all tumors obtained from the children and adolescent and young adult (AYA) groups stained positively for nuclear ABC. Collectively, these results underscore that there are significant correlations between positive ABC nuclear staining and tumor stage and age of patients. These findings support prospective studies asking the question “can positive nuclear ABC staining serve as a putative biomarker of aggressive disease in children and AYA with osteosarcoma?”

In conclusion, the findings in our study support that ABC is associated with and is significant for OS progression/metastatic potential *in vitro* and may potentially serve as a biomarker of aggressive disease. However, further studies with a larger sample size and including clinical outcomes with reference to metastasis or local progression are needed to conclusively determine the value of ABC as a biomarker for aggressive OS in patients.

## MATERIALS AND METHODS

### Cell lines and culture conditions

The SaOS2-LM7 and its parent cell line SaOS2 were a kind gift from Dr. Eugenie Kleinerman, The University of Texas M.D. Anderson Cancer Center, USA. HOS (Catalog no. CRL-1543) and HOS-143B (Catalog no. CRL-8303) were purchased from ATCC. All cell lines were cultured in Minimal Essential Medium (MEM) (Catalog no. 10320-021, Gibco), supplemented with 10% fetal bovine serum (FBS) (Catalog no. 12483-020, Gibco), 1% penicillin-streptomycin (Catalog no. 15140-122, Gibco), 1mM sodium pyruvate (Catalog no. 11360-070, Gibco) and 2mM L-Glutamine (Catalog no. 25030-081, Gibco) at 37°C and 95% O2 and 5% CO2.

### Conditioned media concentration and gelatin zymography

Cells were grown in 6 well plates to 80% confluence, at which time complete media were replaced with 500ul serum free media and incubated for 24 hours. After incubation, the conditioned media were transferred to a Centricon filter (Catalog no. UFC501024, Millipore) and concentrated according to the manufacturer's protocol. 10ul of the concentrated conditioned media in 6X loading buffer were loaded onto an 8% SDS-PAGE containing 2 mg/ml gelatin substrate (Catalog no. G8150, Sigma Aldrich). Conditioned media from HT1080 fibrosarcoma cells was used as a positive control. At completion of electrophoresis, gels were washed in 2.5% Triton X-100 v/v in water for 3 times, 20 minutes each. The washed gels were incubated overnight in incubation buffer (composed of NaCl, CaCl2, Tris and NaN3) at 37°C and then stained with 0.05% Coomassie Brilliant Blue G-250 (Catalog no. B 1131, Sigma) for 2 hours. The gels were then de-stained using aqueous 4% methanol: 8% acetic acid and imaged using the Bio-Rad Gel Doc apparatus and Quantity One software.

### Transwell® Invasion Assay

For cell invasion assay, a Transwell^®^ unit (8 μM) coated with BD Matrigel Basement Matrix was used. The chamber was placed in 24-well plate. SaOS2, SaOS2-LM7, HOS, HOS-143B and MCF-7 cells were counted (40,000-50,000) and seeded in the upper compartment of the chamber. MCF-7 was used as a negative control and an empty chamber with no cells was used as a blank. The cells were incubated in 0.1% FBS DMEM for 24 hours at 37°C and 5% CO_2_. DMEM supplemented with 10% FBS was added to lower compartment of the well to act as a chemoattractant. After completion of incubation period, the chamber was removed from well and cells present on upper compartment of chamber were removed using cotton swab. For cells which were able to invade to lower compartment of chamber, they were washed with 1X PBS and fixed with ice-cold 100% methanol (−20°C) at room temperature for 20 minutes. The cells were then stained with 0.5% crystal violet for 15-20 minutes. For counting the cells, the chamber was analyzed using 10X High Content Microscope and MetaExpress software.

### Preparation of whole cell lysate

Cells were grown to 80% confluence in a 100 mm dish, washed with 1X PBS (Catalog no. SH3025601, Thermo Scientific) and trypsinized in 0.25% Trypsin-EDTA 1X (Catalog no. 25200-056, Gibco). 1x10^6^ cells were lysed using 100 μl of lysis buffer [10mM Tris-HCl pH 7.5, 1mM EDTA, 1mM EGTA, 1% Triton X-100, 2mM phenylmethylsulfonyl fluoride (PMSF), 80ng/ml aprotinin, 40ng/ml chymostatin, 40ng/ml antipain, 40ng/ ml leupeptin, 40ng/ml pepstatin] per 10^6^ cells on ice for 30min. Cellular debris and nuclei were removed by centrifugation.

### Nuclear/cytoplasmic fraction isolation

The nuclear and cytoplasmic fractions were isolated using the NE-PER nuclear/cytoplasmic extraction kit (Catalog no. 78833, Thermo Scientific) according to the manufacturer's protocol using 1x10^6^ cells. The volume of each reagent that was used for isolation was; CER1 – 200 μl, CERII – 11μl, NER – 50 μl. An additional wash step was incorporated to the protocol to reduce the contamination between fractions. After collecting the cytoplasmic fraction, the pellet was re-suspended (tap mixing) in 500 μl of ice cold 1X PBS and centrifuged at 4°C for 5 minutes at maximum speed. The supernatant was carefully discarded, and the pellet was used for isolating the nuclear fraction according to manufacturer's protocol.

### Immunoblotting

Protein quantification of samples were carried out using Pierce^TM^ BCA (Bicinchoninic acid) protein assay kit (Catalog no. 23227, Thermo Scientific). The whole cell lysate samples were prepared by boiling 40 μg of protein in 1X loading buffer for 5 minutes. For the nuclear cytoplasmic fractions, 36 μl of each fraction was boiled with 1X loading buffer for 5 minutes and then placed on ice for 5 minutes. The samples were run on a 7.5% SDS-PAGE and proteins were transferred to a polyvinylidene fluoride (PVDF) membrane (Catalog no. 1620177, Bio-Rad) at 110 V for 70 minutes at 4°C. Blocking of membrane was carried out for 1 hour using 5% non-fat dry milk powder in 1x TBS (Tris buffered saline) containing 0.1% Tween-20 (TBST), followed by incubation of the membrane in primary antibody overnight at 4°C. The membranes were washed 3 times in TBST and then incubated in corresponding Horseradish peroxidase (HRP) linked secondary antibody for 1 hour at room temperature. After secondary antibody incubation, the blots were washed 3 times in TBST for 10 minutes each and visualized using SuperSignal West Femto (Catalog no. 34095, Thermo Fisher) or Western Lighting Plus ECL (Catalog no. NEL104001, Perkin Elmer).

The following antibodies were purchased commercially and used at the indicated dilutions: β-catenin (Catalog no. 9587S, Cell Signaling) 1:1000; Anti-Active-β-catenin (Catalog no. 05-665, Millipore) 1:500; β-actin (Catalog no. sc69879, Santa Cruz) 1:10000; α/β-tubulin (Catalog no. 2148; Cell Signaling) 1:1000; Lamin-B1 (Catalog no. MABE622, Millipore Sigma) 1:1000; Anti-mouse IgG (Catalog no. NA934V, GE Healthcare); Anti-rabbit IgG (Catalog no. NA931V, GE Healthcare) 1:10000.

### Immunofluorescence

Cells were grown to 30-40% confluence on coverslips. Cells were briefly washed with 1x PBS twice and fixed with 4% formaldehyde for 15 minutes at room temperature. Subsequently, cells were permeabilized with 100% methanol at −20°C for 10 minutes and blocked for 1 hour with 5% goat serum (Catalog no. 9023, Sigma Aldrich) in 1x PBS-Triton (0.3%). Cells were then incubated with 1:200 anti-β-catenin antibody (Catalog no. 2677, Cell Signaling) or 1:200 Anti-Active-β-catenin (ABC) diluted in blocking buffer overnight at 4°C. This was followed by incubation with AlexaFluor® 555 goat anti-mouse antibody (Catalog no. A21422, Invitrogen) for visualization. At the completion of secondary antibody incubation, cell nuclei were stained with 300 nM 4′, 6-diamidino-2-phenylindole (DAPI) for 7 minutes (Catalog no. D1360, Invitrogen). Coverslips were briefly rinsed with PBS and mounted on glass slide using Prolong antifade (Catalog no. P7481, Invitrogen). Washes were carried out 3 times, 5 minutes each with PBS after fixation, permeabilization and primary antibody incubation. Imaging was carried out at 40X magnification (oil immersion) using Carl Zeiss Laser Scanning Microscope and image processing was carried out using LSM image browser software.

### High content microscopy

Cells were cultured in 96 well plate (Catalog no. 655090, Greiner Bio one) to 70% confluence. Cell staining was carried out using the immunofluorescence protocol as described above. Images were taken at 10X (NA 0.3) magnification using an automated, high content screening system, ImageXpress Micro XLS, Molecular Devices (USA). Briefly, a defined number of images (10X) were taken per well and the resulted images were stored in a data storage server and analyzed using a predefined multi-wavelength cell scoring algorithm in MetaXpress software package which segments and measures the fluorescence intensities on a per cell base. We measured Cy3 fluorescence intensities from the nuclear and the total cellular region for all the cells in the images. The nuclear region was defined by the part of the cell nuclear label signal of DAPI using a combination of criteria of minimum staining intensity, minimum sizes and maximum sizes of the nucleus. Using the detected nuclear label as a mask, the average intensity of the Cy3 over the nuclear region was obtained. Similarly, the average intensity of Cy3 fluorescence of each cell (both nuclear and cytoplasmic areas) was obtained similarly using the cellular scoring algorithm which detects the whole cell region based on a combination of criteria of nuclear label, Cy3 label intensity as well as minimum and maximum sizes of the cell. The procedure measures Cy3 intensity coming from each cell and provides a quantified average cellular intensity of Cy3 for the total population of cells/ nuclei.

### Quantitative real-time PCR

Total RNA was isolated using RNeasy® Mini Kit (QIAGEN) as per the manufacturer's protocol. 1μg of total RNA was used for reverse transcription of Oligo (dT) (Invitrogen) and Superscript III reverse transcription (Vilnius, LT-02241, Applied Biosystems). Real time quantification of Cyclin D1, VEGF-A, MMP-2, MMP-9 and Axin2 were assessed using power SYBR Green PCR Master Mix (Applied Biosystems). GAPDH was used as the endogenous control. Samples were amplified with a precycling hold at 95˚C for 15 seconds, 30 cycles of annealing and extension at 60 ˚C for 1 minute. The following primers were used: Cyclin D1: sense (5`-CAT CTA CAC CGA CAA CTC CAT C-3`); Cyclin D1: anti-sense (5`-TCT GGC ATT TTG GAG AGG AAG −3`); VEGF-A: sense (5`-AGT CCA ACA TCA CCA TGC AG-3`); VEGF-A: anti-sense (5`-TTC CCT TTC CTC GAA CTG ATT T-3`); MMP-2: sense (5`-GGC CCT GTC ACT CCT GAG AT −3`); MMP-2: anti-sense (5`-GGC ATC CAG GTT ATG GGG GA-3`); MMP-9: sense (5′-CGA ACT TTG ACA GCG ACA AG-3′); MMP-9: anti-sense (5′-CAC TGA GGA ATG ATC TAA GCC C-3′); Axin2: sense (5′-GCGTGGCCAGTCAGCAGAGG-3′); Axin2: anti-sense (5′-CCTGGAGCGCGTGGACACTT-3′); GAPDH: sense (5`-TCA ACG ACC ACT TTG TCA AGC TCA-3`); GAPDH: anti-sense (5`-GCT GGT GGT CCA GGG GTC TTA CT-3`). Each measurement was performed in triplicate with LightCycler@96 (ROCHE) and LightCycler@96 Software. Gene expression was determined using the relative standard curve method normalized to GAPDH-binding protein expression. Histograms are reported as fold change of control which was set at 1.

### Tissue immunohistochemistry

The Osteosarcoma TMA were purchased from Folio Biosciences (Catalog no. ARY-HH0085) and consisted of 40 unique cores in duplicates. Information about each patients’ age, sex, disease pathology and tumor stage were available. Antibodies used for staining included Anti-Active-β-catenin (Catalog no. 05-665, Millipore) and Anti-β-Catenin Clone 14/Beta-Catenin (Catalog no. 610154, BD Biosciences). Colon cancer and spleen tissue were used as positive and negative controls respectively (obtained from Dr. Judith Hugh, Department of Laboratory Medicine& Pathology, University of Alberta). Formalin-fixed, paraffin embedded osteosarcoma tissue slides were obtained from Dr. Atilano Lacson, Department of Laboratory Medicine & Pathology, University of Alberta.

For deparaffinization and rehydration, TMA or tissue slides were baked at 60 degrees for 2 hours, immersed in xylene (2X) for 2 minutes followed by graded EtOH including 100% (2X), 95%, 85%, 75% and 50%, respectively, for 2 minutes each and then ddH2O for 5 minutes. Antigen presentation was carried out by immersing slides in boiling sodium citrate (10mM) for 30 minutes. The slides were washed 3X with wash solution (1X PBS with 0.05% Triton X-100) for 5 minutes each after each step starting from antigen presentation till after ABC solution incubation. Thereafter, slides were blocked for 2 hours in blocking buffer (1X PBS, 5% goat serum, 0.2% Triton X-100, 0.1% BSA and ddH_2_O) followed by overnight incubation with primary antibody (1:200 dilution) at 40C. Following primary antibody incubation, slides were incubated with 0.3% H_2_O_2_ for 30 minutes and then incubated with HRP-labelled secondary antibody (Catalog no. NEF822001EA, Perkin Elmer) for 2 hours. For signal amplification, slides were treated with Tyramide Signal Amplification (Catalog no. NEL700A001KT, Perkin Elmer) reagent and Avidin-Biotin Complex solution (Catalog no. PK-6100, Vector Laboratories) for 7 and 30 minutes respectively. After this, they were immersed in DAB chromogenic substrate (Catalog no. SK-4105, Vector Laboratories) for 1-10 minutes until a brown stain was detected and then washed under running tap water for 5 minutes. This was followed by hematoxylin (Catalog no. SH26-500D, Fisher Scientific) staining for 30 seconds followed by incubation with Scott's Top water (3.5g sodium bicarbonate, 20g magnesium sulphate and ddH_2_O). Consequently, the slides were dehydrated in graded EtOH including 50%, 75%, 85%, 95% and 100%, respectively, for 2 minutes each, followed by xylene for 2 minutes. Coverslips were then mounted on slides using Permount (Catalog no. SP15-100, Fisher Scientific).

The TMA was scored by two pathologists who were blinded to all patient information. The cores in the TMA were scored based on the percentage of positive nuclear ABC staining cells in each core [(number of cells positive for nuclear ABC staining/total number of cells) ^*^100]. The scoring was assigned as follows: 0 (no nuclear staining); 1+ (≤30% of positive nuclear staining cells); 2+ (31-60% of positive nuclear staining cells) and 3+ (>60% of positive nuclear staining cells.

### TCF/LEF transcriptional activity

HOS/HOS-143B and SaOS2/SaOS2-LM7 cell lines were cultured in 100 mm plate to 80% confluence. TCF/LEF induced transcriptional activity was determined by using a TCF/LEF promoter-Luciferase reporter construct, pTOPFlash, by techniques reported previously [48] and according to the manufacturer's instructions (Promega E1500). Briefly, a luciferase reporter assay was performed whereby cells (HOS and HOS-143B) were transfected using Lipofectamine 3000 (Catalog no. L3000008, Invitrogen) with TCF promoter/luciferase reporter gene (pTOPFlash) and subsequently treated with 10mM LiCl (for the activation of Wnt/β-catenin signaling). The TOPFlash-luciferase reporter construct specifically measures β-catenin-ABC/TCF regulated transcriptional function and is comprised of a multimeric synthetic β-catenin/TCF-4 binding site upstream of a Thymidine Kinase (TK) minimal promoter and a Luciferase open reading frame. A mutated TCF-Luciferase reporter construct (pFOPFlash) served as a negative control for TOPFlash activity. The reporter activity ratio was measured using a luminometer (Fluor Star OMEGA: BMG Labtech).

### Statistics

A two-sided Student's *t*-test (GraphPad PRISM Software; GraphPad Software, Inc., CA, USA) was used compare differences between groups. Results are presented as mean ± SD and values with *P* < 0.05 were considered statistically significant. Correlation between patients’ gender and nuclear ABC staining was done using Fisher Exact test. Statistical analysis to compare tumors with positive/negative nuclear ABC staining with patient age was done using non-parametric Mann-Whitney test. Association between stage of tumor and positive nuclear ABC staining was analyzed using Kruskal Wallis Trend test.

## Abbreviations

OS: Osteosarcoma
ABC: Activated β-Catenin
TMA: Tumor Tissue Microarray
